# Genome Sequence of Canine Adenovirus Type 1 Isolated from a Wolf (*Canis lupus*) in Southern Italy

**DOI:** 10.1128/genomeA.00225-17

**Published:** 2017-04-20

**Authors:** Federica Pizzurro, Maurilia Marcacci, Guendalina Zaccaria, Massimiliano Orsini, Francesca Cito, Alfonso Rosamilia, Ludovica Di Renzo, Daniela Malatesta, Daria Di Sabatino, Alessio Lorusso

**Affiliations:** a Dipartimento di Scienze Veterinarie, Università di Pisa, Pisa, Italy; b Virology Unit, Istituto Zooprofilattico Sperimentale dell’Abruzzo e Molise (IZSAM), Teramo, Italy

## Abstract

Canine adenovirus type 1 (CAdV-1), a DNA virus of the family *Adenoviridae*, causes infectious canine hepatitis, a highly contagious disease primarily affecting canids. In this report, we describe the isolation and whole-genome sequence of a CAdV-1 isolate from the liver of a free-ranging wolf (*Canis lupus*).

## GENOME ANNOUNCEMENT

Infectious canine hepatitis (ICH) is a highly contagious disease, affecting primarily canids, caused by canine adenovirus type 1 (CAdV-1), a nonenveloped icosahedral double-stranded DNA virus that belongs to the genus *Mastadenovirus* of the family *Adenoviridae* (1). Transmission of CAdV-1 occurs through direct contact or exposure to infectious saliva, feces, urine, or respiratory secretion (2). Lesions in dogs include severe necrotizing hepatitis, disseminated intravascular coagulation, and vasculitis, the latter manifesting as encephalitis and glomerulonephritis, caused by viral replication in hepatocytes and vascular endothelial cells (3). Infection by CAdV-1 has also been documented in wild species, including foxes, wolves, bears, and coyotes (4, 5). In September 2015, a 4-month-old male wolf (*Canis lupus*) was found at the edge of a road with multiple fractures in the province of Campobasso (Molise region, southern Italy). The wolf was referred to the regional wildlife rescue center of the Italian State Forestry Corps, where it died 2 days later; the wolf was then necropsied at IZSAM. Histologically, multifocal coagulative necrosis of hepatocytes was recorded. Many hepatocytes were devoid of nuclei, while others showed vacuolation in the cytoplasm suggestive of fatty change. Basophilic intranuclear inclusions were revealed in hepatocytes adjacent to necrotic areas. 

DNA extracted from liver homogenate tested positive for CAdV-1 by conventional PCR (6); the homogenate was subsequently used for viral isolation on MDCK cells. CAdV-1 ITL2015 was successfully isolated at the third cell passage. Other key pathogens of canids were not detected by molecular assays. Genomic DNA was extracted from the cell culture supernatant (DNeasy blood and tissue kit, Qiagen) and quantified with the Qubit DNA HS assay kit (Thermo Fisher Scientific) for whole-genome sequencing. One nanogram of DNA was used for library preparation using the Nextera XT library prep kit (Illumina). Deep sequencing was performed on the NextSeq 500 using the NextSeq 500/550 mid-output reagent cartridge version 2 with 2,300 cycles and standard 150-bp paired-end reads (Illumina). A total of 996,302 paired-end reads were obtained. Reads were trimmed and assembled using a *de novo* approach with SPAdes version 3.1.0, followed by manual finishing. The mean coverage of the obtained consensus sequence (30,531 bp) was 1,423×. Genome comparison demonstrated high nucleotide identity (up to 100%) with either whole or partial sequences of CAdV-1 available in the GenBank database, including partial Italian sequences. 

To our knowledge, this is the first report which describes the isolation and whole-genome sequencing of CAdV-1 from a free-ranging wolf. Even if CAdV-1 has been effectively controlled in Italy by vaccination, it is still circulating in dogs; clinical outbreaks have indeed been recently recorded in owned dogs, animal shelters, and breeding kennels with severe and fatal outcomes (7, 8). Further studies are needed to assess the distribution of CAdV-1 in the wild population. Currently, vaccination of domestic dogs for CAdV-1 is still the only valid strategy to prevent clinical disease in dogs and transmission from dogs to wildlife and vice versa.

### Accession number(s).

The nucleotide sequence of CAdV-1 ITL2015 has been deposited in GenBank under the accession number KX545420.
